# Survival of bronchopulmonary cancers according to radon exposure

**DOI:** 10.3389/fpubh.2023.1306455

**Published:** 2024-01-24

**Authors:** Juliette Dessemon, Olivia Perol, Cécile Chauvel, Hugo Noelle, Thomas Coudon, Lény Grassot, Nicolas Foray, Elodie Belladame, Jérôme Fayette, Françoise Fournie, Aurélie Swalduz, Eve-Marie Neidhart, Pierre Saintigny, Mayeul Tabutin, Maxime Boussageon, Frédéric Gomez, Virginie Avrillon, Maurice Perol, Barbara Charbotel, Béatrice Fervers

**Affiliations:** ^1^Département Prévention Cancer Environnement, Center Léon Bérard, Lyon, France; ^2^Faculté de Médecine Lyon Est, Université de Lyon, Lyon, France; ^3^Inserm UMR1296, “Radiation: Defense, Health Environment,” Center Léon Bérard, Lyon, France; ^4^Center of Excellence in Respiratory Pathogens (CERP), Hospices Civils de Lyon, Lyon, France; ^5^Centre International de Recherche en Infectiologie (CIRI), Inserm U1111, CNRS UMR5308, ENS de Lyon, Université Claude Bernard Lyon 1, Lyon, France; ^6^Département de Cancérologie Médicale, Center Léon Bérard, Lyon, France; ^7^Département Interdisciplinaire de Soins de Support du Patient en Oncologie, Center Léon Bérard, Lyon, France; ^8^Département de Chirurgie Cancérologique, Center Léon Bérard, Lyon, France; ^9^Département de Santé Publique, Center Léon Bérard, Lyon, France; ^10^Université de Lyon, Université Lyon 1, Université Gustave Eiffel-Ifsttar, Umrestte, UMR, Lyon, France; ^11^CRPPE-Lyon, Center Régional de Pathologies Professionnelles et Environnementales de Lyon, Center Hospitalier Lyon Sud, Hospices Civils de Lyon, Lyon, France

**Keywords:** radon, lung cancer, survival, environmental exposure, public health

## Abstract

**Introduction:**

Residential exposure is estimated to be responsible for nearly 10% of lung cancers in 2015 in France, making it the second leading cause, after tobacco. The Auvergne-Rhône-Alpes region, in the southwest of France, is particularly affected by this exposure as 30% of the population lives in areas with medium or high radon potential. This study aimed to investigate the impact of radon exposure on the survival of lung cancer patients.

**Methods:**

In this single-center study, patients with a histologically confirmed diagnosis of lung cancer, and newly managed, were prospectively included between 2014 and 2020. Univariate and multivariate survival analyses were carried out using a non-proportional risk survival model to consider variations in risk over time.

**Results:**

A total of 1,477 patients were included in the analysis. In the multivariate analysis and after adjustment for covariates, radon exposure was not statistically associated with survival of bronchopulmonary cancers (HR = 0.82 [0.54–1.23], HR = 0.92 [0.72–1.18], HR = 0.95 [0.76–1.19] at 1, 3, and 5 years, respectively, for patients residing in category 2 municipalities; HR = 0.87 [0.66–1.16], HR = 0.92 [0.76–1.10], and HR = 0.89 [0.75–1.06] at 1, 3, and 5 years, respectively, for patients residing in category 3 municipalities).

**Discussion:**

Although radon exposure is known to increase the risk of lung cancer, in the present study, no significant association was found between radon exposure and survival of bronchopulmonary cancers.

## 1 Introduction

Lung cancer remains one of the leading causes of cancer-related mortality worldwide, with 37,095 deaths recorded in France in 2020 (1). While tobacco is by far the most important etiological factor in lung cancer (2, 3), responsible for 81% of all new lung cancer cases (2), radon is the leading risk factor in non-smokers and the second leading cause in smokers (3).

Radon is a naturally occurring radioactive gas resulting from the decay of radium and uranium in the earth’s crust. If inhaled with airborne particles, it can cause cells in the respiratory tract to become irradiated by alpha-particles, leading to an increased risk of cancer. The International Agency for Research on Cancer (IARC) has recognized radon as a definite carcinogen of lung cancer since 1988 (4). Although radon does not pose a risk outdoors due to its dilution, it can be hazardous when it accumulates in confined spaces, particularly in private homes (5). Residential exposure to radon is a significant public health concern in France. In public buildings located in areas with significant radon potential exposure, measurements are mandatory and the average reference level for radon exposure has been set at 300 Bq/m^3^ per year since 2018 (6). It has been estimated that residential radon exposure in France accounts for 9.8% of all lung cancers (4,000 cases) (7) and approximately 10% of all lung cancer deaths (3,000 deaths) in 2015 (8).

Despite the established link between radon exposure and the development of lung cancer, the impact of this exposure on the prognosis and survival of lung cancer patients has received little attention. A study conducted in Spain among 369 non-smoking lung cancer patients reported a significant decrease in survival at 5 years in populations exposed to high levels of residential radon (>300 Bq/m^3^) (9).

This study aimed to delve deeper into the relationship between radon exposure and survival in lung cancer patients. By using survival analysis, we sought to further understand how exposure to radon impacts the prognosis of individuals diagnosed with lung cancer. Ultimately, our goal was to provide valuable insights that could improve prevention in lung cancer patients and survivors.

## 2 Materials and methods

### 2.1 Study population

This monocentric study was conducted at the Center Léon Berard (CLB), a comprehensive cancer center in Lyon, France, affiliated with the National Federation of Cancer Centers Unicancer (10). Patients included in the study had histologically confirmed lung cancer, were treated at the CLB, and were prospectively integrated in the PROPOUMON (11, 12) database between March 25, 2014 and August 31, 2020. Non-inclusion criteria were residence outside the Auvergne-Rhône-Alpes region, unknown addresses, missing data for the covariates, and refusal to participate in the study.

The study protocol was approved by the Center Léon Berard Committee of Research Ethics (reference 2022–003). The study database was reported to the National Commission for Data Protection and Liberties (CNIL; reference number: 2016177 v0).

### 2.2 Data collection

Information concerning patients’ baseline demographics and clinical characteristics was retrieved from the medical records. The data collected included sex at birth, smoking status at diagnosis, histologic subtype, cancer stage at diagnosis (TNM), presence of cerebral metastases, and gene mutations. In this study, patients who were quitting or had quit smoking less than 1 week prior to diagnosis were classified as active smokers. Staging of lung cancer was based on the 8th American Joint Committee on Cancer TNM classification (13). TNM stages at diagnosis were grouped into three categories (stage I and II grouped together, stage III, and stage IV with and without brain metastasis). Histologic types were categorized into non-squamous non-small cell lung cancer (non-squamous NSCLC, including adenocarcinoma and not otherwise specified carcinoma), squamous non-small cell lung cancer (squamous NSCLC), and small cell lung cancer (SCLC). Patients with neuroendocrine histological type other than small cells (*n* = 39) were excluded from the analyses (14). *Standard* of *living* (i.e., census-based median household disposable income for 2020) at the municipality level [French National Institute for Statistics and Economic Studies (INSEE)] was used as an area-level surrogate measure of individual socio-economic status (15). The variable was dichotomised using the median standard of living of the Auvergne-Rhône-Alpes region (i.e., 22480€ in 2018).

### 2.3 Assessment of radon exposure

The assessment of the radon exposure of the study participants was based on the geogenic radon potential of their municipality of residence, using the postal code of residence from the patients’ medical records. The geogenic radon potential of French municipalities was determined by the Institute for Radiological Protection and Nuclear Safety (IRSN) (16, 17). Based on the mapping of multiple radon-related spatialized variables, including the uranium content of the rocks and the presence of geological factors, such as faults, underground mining structures, and hydrothermal spring sites, which may facilitate radon transport, IRSN classified French municipalities into three categories according to the radon release potential. Category 1 municipalities are located entirely on geological formations with low uranium contents and no geological factors that may facilitate radon transport. Category 2 municipalities are also located on geological formations with low uranium contents but present, for at least part of their surface are, geological factors that can facilitate the passage of radon to the surface. Category 3 municipalities are those that have geological formations with higher estimated uranium contents, at least for part of their surface area. According to the national measurement campaign by IRSN, 20% of buildings exceed 100 Bq/m^3^ and less than 2% exceed 300 Bq/m^3^ in category 1 municipalities, whereas more than 40% of the buildings located in category 3 municipalities exceed 100 Bq/m^3^ and more than 10% exceed 300 Bq/m^3^. The average radon concentration in French homes is 68 Bq/m^3^. The Auvergne-Rhône-Alpes region in south-eastern France, with an area of 69,711 km^2^ and comprising 12 departments (EU NUTS 3-BIS) (18), is particularly affected by radon exposure, with 30% of its population estimated to reside in areas with medium to high radon potential (Categories 2 and 3) (19).

### 2.4 Follow-up

The follow-up time was defined as the period between diagnosis and the date of last follow-up or date of death recorded in the medical record. If the date of last follow-up was more than 6 months from the point date of March 31, 2021, the vital status of the patient was verified using the national death registration file. The date of the last follow-up was set to the date of death if the patient was matched to the national death registration file; otherwise, the date of last follow-up was set to the point date. The last medical record review was conducted in October 2022 and focussed on checking and collecting the dates of the latest news.

### 2.5 Statistical analyses

We conducted a univariate descriptive analysis of the study population according to the radon potential category of their municipality of residence. Continuous variables were reported as median and interquartile range (IQR). Categorical variables were presented as numbers of patients in each category and their frequencies. Comparisons on patients’ characteristics were performed using Pearson’s chi-squared test and Kruskal–Wallis rank sum test. Kaplan–Meier estimates of the survival for each radon exposure category were plotted (20).

The standard approach for analyzing time-to-event data is to use the Cox model (21). This model applies if all covariate effects are constant over time, indicating that hazard ratios are proportional. The statistical test for goodness-of-fit of the Cox model implemented in the *cox.zph* function of the *survival* R package indicated violations of the proportional hazards assumption for most variables for overall survival (22) (Supplementary material 1). We chose to deal with non-proportional hazards by allowing the coefficients (corresponding to hazard ratios) to vary over time (23–25).

The analyses were conducted with the *timecox* function of the *timereg* R package, which implements a semi-parametric multiplicative model (26, 27). In this model, regression coefficients can either be fixed or vary over time, allowing more flexibility than the Cox model. For each covariate, the resulting estimate of the regression coefficient is either a scalar or a non-parametric function of time. Furthermore, this method is appealing as a graphical representation of the time-dependent coefficients is provided to visually detect departure from fixed effects (see Supplementary material 2). A Cramer–von Mises goodness-of-fit test enables us to determine whether the covariate is to be included with a fixed (*p* < 0.05) or a time-dependent (*p* > 0.05) regression effect.

Covariates included in the survival analyses were age at diagnosis, sex, smoking status, histological type, tumor stage at diagnosis, and gene mutations (EGFR, BRAF, KRAS, HER2, ALK, and ROS 1), as well as area-level living standard. Univariate analyses were performed separately for each covariate. Their effect was set as either constant or time-varying according to the Cramer–von Mises test. Then, multivariable analyses were considered. A backward selection procedure was performed to include significant covariates in the model, using the Supremum test (*p* < 0.05) (26). Gene mutations were not statistically associated with survival (*p* > 0.05) and were not integrated in the final model. Once the variable selection was completed, the remaining step was the choice between fixed or time-varying effects for each covariate. First, all covariates were included with time-varying effects. Recursively, in a backward procedure, effects were turned into fixed effects using the Cramer–von Mises test. In the end, all covariates with time-varying effects led to significant Cramer–von Mises tests (*p* < 0.05; Supplementary material 3). All analyses were also performed without the area-level standard of living as an explanatory variable, leading to the same results (not shown here).

The results are presented as mean hazard ratios (HR) and their 95% confidence intervals (CI). Hazard ratios were obtained by dividing the cumulative risk by the number of elapsed months. Three separate analyses were conducted to study the survival of lung cancer patients until either 12 months (1 year), or 36 months (3 years) or 60 months (5 years). These durations were chosen to allow consistency and comparison of the results between different studies on lung cancer survival already carried out (1, 3, and 5 years being the cut-off points chosen in most articles). In addition, this limited the lack of power in the follow-up time due to patients’ deaths and censoring. All statistical analyses were performed with R statistical software (R version 4.0.3).

## 3 Results

**Figure 1:**
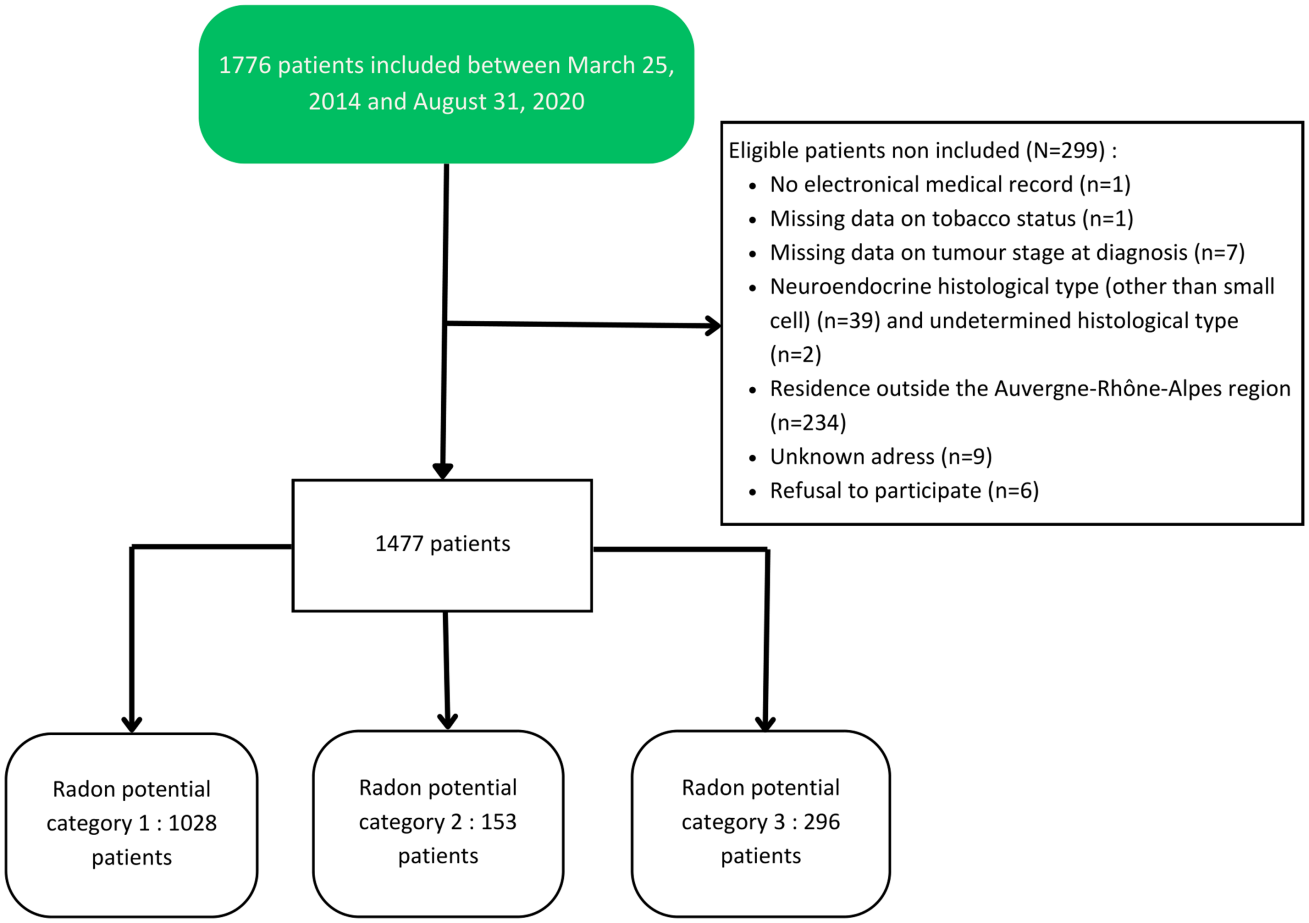
Flowchart of included patients.

**Figure 2:**
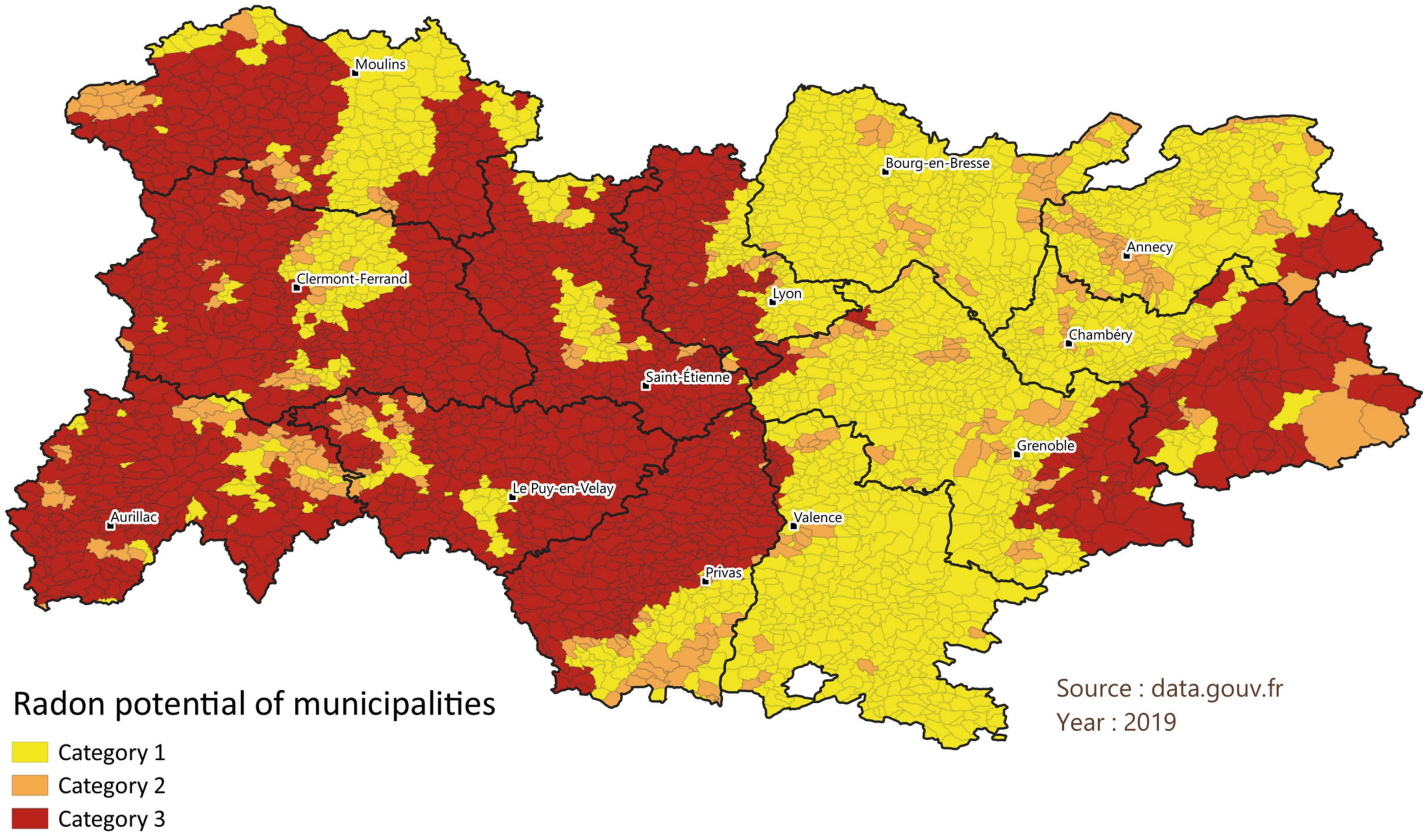
Radon release potential from soil in the municipalities of Auvergne-Rhône-Alpes in 2019.

A total of 1,477 patients residing in the Auvergne-Rhône-Alpes region were included between 2014 and 2020: 1,028 lived in category 1 municipalities, 153 in category 2, and 296 in category 3 (Figures 1, 2). Demographic and clinical characteristics are presented in Table 1. Almost two-thirds of the included patients were males (64%), and the median age was 65 years [IQR 56–71]. More than half of the patients included lived in municipalities with an area-based standard of living above the regional median (54%). At diagnosis, half of the patients (51%) were former smokers and 30% were current smokers. Non-small cell lung cancer (NSCLC) was the main histological type (72%). Half of the patients (53%) had a stage IV tumor when they were treated at the Center Leon Berard, of which 17% had cerebral metastases. No statistical differences were observed between the three radon categories on age at diagnosis, sex, area-level living standard, smoking status at diagnosis, histological type, and tumoral stage at diagnosis (Table 1). A total of 68% of the included patients died. At 1 year of follow-up, 1,142 patients were still alive (77%). This number dropped to 716 (48%) and 595 (40%) at 3 and 5 years of follow-up, respectively. The median length of follow-up was 29 months [IQR 13–51] and was similar between the three groups with 28 months [IQR 13–49], 30 months [IQR 17–52], and 29 months [IQR 13–53] for patients residing in municipalities of category 1, 2, and 3, respectively. Median overall survival was 33 months [IQR 30–36] and 31 months [IQR 28–34] for patients in potential radon category 1, 38 months [IQR 29–51] for patients in category 2, and 38 months [IQR 29–50] for patients in category 3.

**Table 1:** Description of the lung patients’ demographic and clinical characteristics according to the category of radon exposure.

		Radon exposure			
Variable	Overall, *N* = 1,477	Category 1, *N* = 1,028	Category 2, *N* = 153	Category 3, *N* = 296	*p* value*^1^*
Median length of follow-up in months (IQR)	29 (13, 51)	28 (13, 49)	30 (17, 52)	29 (13, 53)	0.30
Median survival (IQR)	33 (30, 36)	31 (28, 34)	38 (29, 51)	38 (29, 50)	0.10
Median age at diagnosis in years (IQR)	65 (56, 71)	65 (56, 72)	63 (55, 69)	64 (56, 70)	0.11
Sex—*N* (%)					0.42
Female	539 (36%)	367 (36%)	63 (41%)	109 (37%)	
Male	938 (64%)	661 (64%)	90 (59%)	187 (63%)	
Area-level living standard—*N* (%)					0.13
Below the regional median	672 (46%)	464 (45%)	61 (40%)	147 (50%)	
Above the regional median	804 (54%)	563 (55%)	92 (60%)	149 (50%)	
Smoking status—*N* (%)					0.17
Never-smokers	279 (19%)	178 (17%)	31 (20%)	70 (24%)	
Former smokers	751 (51%)	530 (52%)	76 (50%)	145 (49%)	
Current smokers	447 (30%)	320 (31%)	46 (30%)	81 (27%)	
Histological type—*N* (%)					0.66
Non-squamous NSCLC	1,059 (72%)	735 (71%)	108 (71%)	216 (73%)	
SCLC	120 (8.1%)	90 (8.8%)	10 (6.5%)	20 (6.8%)	
Squamous NSCLC	298 (20%)	203 (20%)	35 (23%)	60 (20%)	
Tumoral stage at diagnosis—*N* (%)					0.86
I/II	354 (24%)	245 (24%)	39 (25%)	70 (24%)	
III	346 (23%)	237 (23%)	40 (26%)	69 (23%)	
IV without brain metastasis	522 (35%)	373 (36%)	46 (30%)	103 (35%)	
IV with brain metastasis	255 (17%)	173 (17%)	28 (18%)	54 (18%)	
Vital status—*N* (%)					0.087
Deceased	1,004 (68%)	717 (70%)	98 (64%)	189 (64%)	
Alive	473 (32%)	311 (30%)	55 (36%)	107 (36%)	

^1^Pearson’s chi-squared test; Kruskal–Wallis rank sum test.

**Figure 3:**
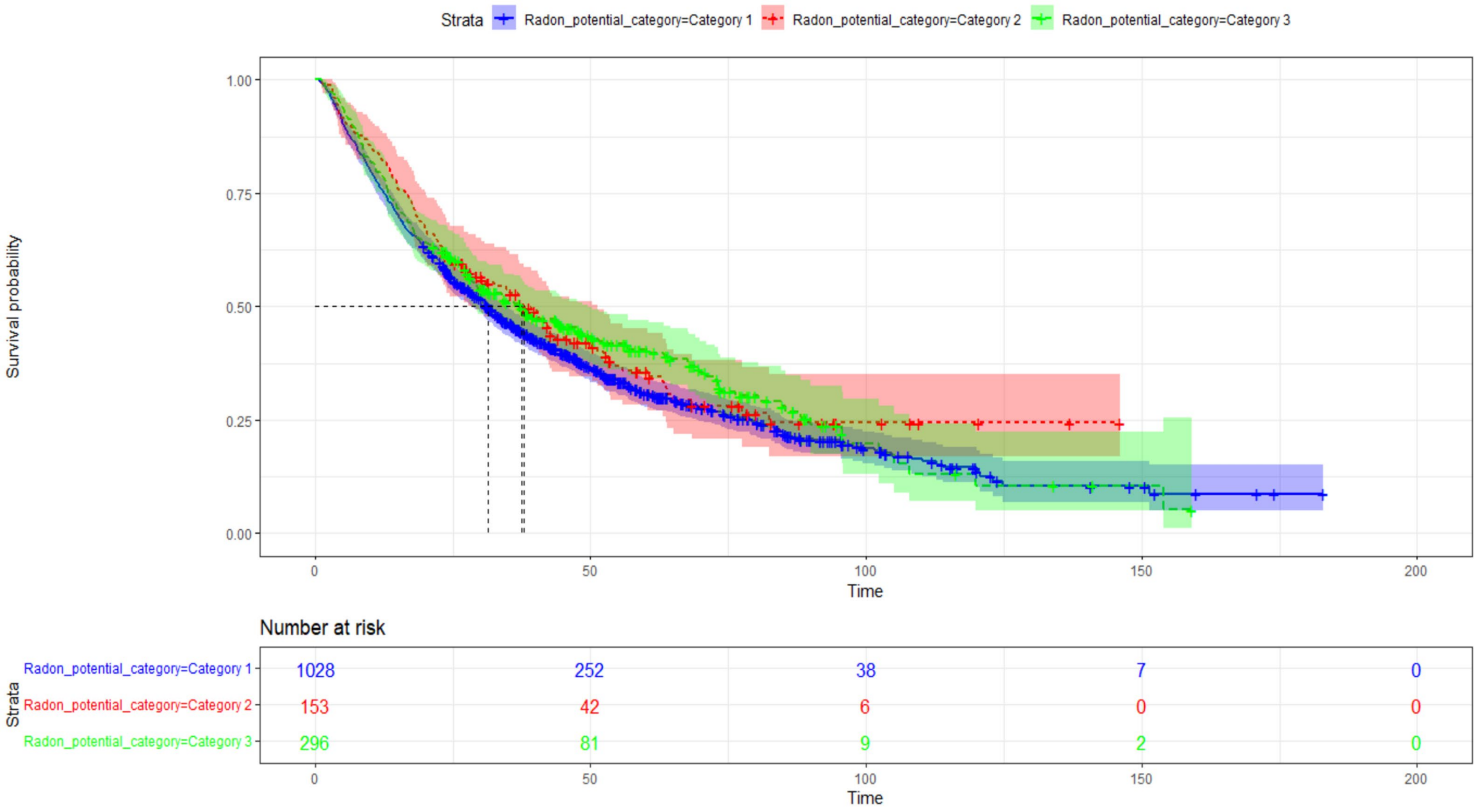
Kaplan–Meier survival curves according to residential radon potential categories.

Figure 3 displays the Kaplan–Meier estimates of the survival for each radon exposure category. All three survival estimates are close and cross at several time points, indicating few discrepancies between the time-to-death of patients according to their exposure to radon. In addition, the crossings of Kaplan–Meier estimates give hints that the effects of radon exposure, if statistically significant, vary over time (non-proportional assumption).

Univariable analyses are presented in Table 2. This analysis showed that age at diagnosis, ever-smokers (former smokers and current smokers), small cell lung cancers, and advanced tumor stage were associated with higher mortality at 1, 3, and 5 years of follow-up. Squamous non-small cell lung cancers were not statistically associated with lower survival except at 3 years of follow-up (HR: 1.20 [CI 1.01–1.44], *p* = 0.03). Area-level living standard and exposure to radon were not statistically associated with survival in lung cancer patients in univariable analyses.

**Table 2:** Univariate analyses of the survival at 1, 3, and 5 years of lung cancer patients according to each demographic or clinical variable separately, under the non-proportional hazards model.

	1-year survival		3-year survival		5-year survival	
	HR [95% CI]	*p* value	HR [95% CI]	*p* value	HR [95% CI]	*p* value
Age at diagnosis (per year)	**1.01 [1.00–1.02]**	***p* < 0.01**	**1.01 [1.00–1.02]**	***p* < 0.01**	**1.01 [1.01–1.02]**	***p* < 0.01**
Sex						
Female	-	-	-	-	-	-
Male	**1.47 [1.16–1.86]**	***p* < 0.01**	**1.24 [1.06–1.44]**	***p* < 0.01**	**1.24 [1.08–1.42]**	***p* < 0.01**
Area-level living standard						
Below the regional median	-	-	-	-	-	-
Above the regional median	0.92 [0.75–1.15]	*p* = 0.51	0.91 [0.79–1.05]	*p* = 0.17	1.12 [0.95–1.31]	*p* = 0.46
Smoking status at diagnosis						
Never smoker	-	-	-	-	-	-
Former smoker	**2.31 [1.58–3.38]**	***p* < 0.01**	**1.61 [1.29–1.99]**	***p* < 0.01**	**1.56 [1.29–1.89]**	***p* < 0.01**
Current smoker	**2.56 [1.74–3.78]**	***p* < 0.01**	**1.73 [1.37–2.18]**	***p* < 0.01**	**1.67 [1.36–2.04]**	***p* < 0.01**
Histologic type						
Non-squamous NSCLC	-	-	-	-	-	-
SCLC	**2.62 [1.75–3.94]**	***p* < 0.01**	**2.49 [2.01–3.10]**	***p* < 0.01**	**2.33 [1.89–2.86]**	***p* < 0.01**
Squamous NSCLC	1.14 [0.87–1.49]	*p* = 0.35	**1.20 [1.01–1.44]**	***p* = 0.03**	1.16 [0.99–1.37]	*p* = 0.06
Stage at diagnosis						
I/II	-	-	-	-	-	-
III	**4.15 [2.44–7.06]**	***p* < 0.01**	**2.13 [1.64–2.76]**	***p* < 0.01**	**2.06 [1.65–2.59]**	***p* < 0.01**
IV without brain metastasis	**5.47 [3.49–8.58]**	***p* < 0.01**	**3.84 [3.05–4.84]**	***p* < 0.01**	**3.26 [2.64–4.02]**	***p* < 0.01**
IV with brain metastasis	**7.24 [4.53–11.59]**	***p* < 0.01**	**4.20 [3.24–5.46]**	***p* < 0.01**	**3.28 [2.59–4.15]**	***p* < 0.01**
Potential radon categories						
Category 1	-	-	-	-	-	-
Category 2	0.71 [0.48–1.06]	*p* = 0.12	0.82 [0.64–1.05]	*p* = 0.09	0.86 [0.69–1.07]	*p* = 0.15
Category 3	0.84 [0.63–1.05]	*p* = 0.24	0.88 [0.73–1.05]	*p* = 0.14	0.88 [0.75–1.05]	*p* = 0.22

Bold values are indicate statistical significance.

In multivariable analyses (Table 3), male sex was statistically associated with lung cancer survival at 1 year (HR: 1.33 [CI 1.04–1.70], *p* = 0.05) and was not statistically associated with survival at 3 and 5 years of follow-up (HR: 1.14 [CI 0.97–1.34], *p* = 0.09 and HR: 1.14 [CI 0.98–1.32], *p* = 0.07, respectively). Area-level living standard above the regional median remained not associated with survival (*p* > 0.05). Other major prognostic factors (age at diagnosis, smoking status, histological type, and tumor stage at diagnosis) remained associated with lower survival in multivariable analyses. After adjustment for these prognostic factors, exposure to radon remained not associated with survival. Hazard ratios for patients residing in municipalities of potential radon category 2 were 0.82 [CI 0.54–1.23] (*p* = 0.37) at 1 year, 0.92 [CI 0.72–1.18] (*p* = 0.49) at 2 years, and 0.96 [CI 0.76–1.19] (*p* = 0.66) at 5 years of follow-up. Hazard ratios for patients exposed to medium- to high-potential radon (category 3) were 0.87 ([CI 0.66–1.16], *p* = 0.40), 0.92 ([CI 0.76–1.10], *p* = 0.34), and 0.89 ([CI 0.75–1.06], *p* = 0.16) for 1, 3, and 5 years of follow-up, respectively.

**Table 3:** Multivariable analyses of the survival at 1, 3, and 5 years of lung cancer patients according to demographic and clinical variables, under the non-proportional hazards model.

	Survival at 1 year of follow-up		Survival at 3 year of follow-up		Survival at 5 year of follow-up	
	HR [95% CI]	*p* value	HR [95% CI]	*p* value	HR [95% CI]	*p* value
Age at diagnosis (per year)	**1.02 [1.01–1.04]**	***p* < 0.01**	**1.02 [1.01–1.03]**	***p* < 0.01**	**1.02 [1.02–1.03]**	***p* < 0.01**
Sex						
Female	-	-	-	-	-	-
Male	**1.33 [1.04–1.70]**	***p* = 0.05**	1.14 [0.97–1.34]	*p* = 0.09	1.14 [0.98–1.32]	*p* = 0.07
Area-level living standard						
Below the regional median	-	-	-	-	-	-
Above the regional median	0.92 [0.74–1.15]	*p* = 0.52	0.89 [0.77–1.03]	*p* = 0.09	1.04 [0.89–1.21]	*p* = 0.37
Smoking status at diagnosis						
Never smoker	-	-	-	-	-	-
Former smoker	**2.35 [1.57–3.53]**	***p* < 0.01**	**1.85 [1.46–2.34]**	***p* < 0.01**	**1.74 [1.42–2.14]**	***p* < 0.01**
Current smoker	**3.10 [2.00–4.81]**	***p* < 0.01**	**2.21 [1.71–2.86]**	***p* < 0.01**	**2.07 [1.65–2.59]**	***p* < 0.01**
Histologic type						
Non-squamous NSCLC	-	-	-	-	-	-
SCLC	**2.13 [1.39–3.27]**	***p* < 0.01**	**2.06 [1.65–2.57]**	***p* < 0.01**	**1.96 [1.58–2.42]**	***p* < 0.01**
Squamous NSCLC	1.29 [0.97–1.71]	*p* = 0.12	**1.46 [1.21–1.76]**	***p* < 0.01**	**1.40 [1.18–1.67]**	***p* < 0.01**
Stage at diagnosis						
I/II	-	-	-	-	-	-
III	**4.18 [2.43–7.17]**	***p* < 0.01**	**2.13 [1.64–2.77]**	***p* < 0.01**	**2.04 [1.62–2.57]**	***p* < 0.01**
IV without brain metastasis	**6.49 [4.10–10.28]**	***p* < 0.01**	**4.76 [3.74–6.05]**	***p* < 0.01**	**3.97 [3.17–4.97]**	***p* < 0.01**
IV with brain metastasis	**9.58 [5.93–15.49]**	***p* < 0.01**	**5.93 [4.53–7.77]**	***p* < 0.01**	**4.20 [3.24–5.43]**	***p* < 0.01**
Potential radon categories						
Category 1	-	-	-	-	-	-
Category 2	0.82 [0.54–1.23]	*p* = 0.37	0.92 [0.72–1.18]	*p* = 0.49	0.95 [0.76–1.19]	*p* = 0.66
Category 3	0.87 [0.66–1.16]	*p* = 0.40	0.92 [0.76–1.10]	*p* = 0.34	0.89 [0.75–1.06]	*p* = 0.16

Bold values are indicate statistical significance.

## 4 Discussion

The present study assessed the impact of residential radon exposure on lung cancer patients’ survival. Results showed that, while major prognostic factors were associated with survival (28–30), no significant association was found with radon exposure in multivariable analysis.

This result differs from that observed in the study of Casal-Mouriño et al. (9) reporting a decrease in survival in non-smoker lung cancer patients exposed to high levels of radon (HR = 1.41 at 3 years of follow-up, *p* = 0.03 and HR = 1.42 at 5 years of follow-up, *p* = 0.02). In study by Casal-Mouriño, radon exposure is based on measurements made directly in the homes of the patients and expressed in becquerels per cubic meter (Bq/m^3^). As indoor radon measurement data were not available in the present study, we assessed radon exposure based on the radon potential categories for the municipality of residence. Although the differences observed between the two studies could be partly explained by the different methodology and scale of the radon exposure assessment, radon geogenic potential is estimated to have the greatest influence on indoor radon concentrations (17) and is considered to be consistent with the results of residential measurement campaigns conducted in France (16).

Our study has several strengths. It is the largest study investigating the impact of radon exposure on survival in lung cancer patients. A large cohort of 1,477 patients, from several urban and rural geographical areas in the region, were included from the CLB with detailed clinical data available allowing us to adjust for covariates significantly associated with lung cancer survival.

Moreover, as the proportional hazard assumption was not found, we did not apply the standard Cox model (24). We analyzed data using an extension of the Cox model in which the regression parameters can vary over time (26). This allows more flexibility and a resulting model better fitted to the data. Particular attention was paid to the study of the variation of the effects over time, in order to have a better fit of the survival model.

However, some limitations should be noted. A key limitation in the present study is the lack of indoor radon measurements to assess exposure. As more than two-thirds of the patients had died, and given the absence of previous studies providing scientific justification, there were ethical concerns to contact the relatives to perform longitudinal indoor radon measurements. To address this limitation, we used the geogenic radon potential as a surrogate for indoor radon exposure based on a previous French national study, having demonstrated close agreement between geogenic radon potential and average indoor radon concentrations (17). Similar correlations have been reported for Sweden (31). In the study by Demoury et al. (17), the geogenic radon potential was found to have the most significant influence on indoor radon concentrations, while different housing characteristics altogether (i.e., building material, year of construction, foundation type, building type, and floor level) only explained 8% of the indoor radon concentration variability. According to the study by Lorenzo-González et al. (32), the height of the story is most strongly associated with indoor radon concentrations. Exposure estimates using the municipality geogenic radon potential without taking into account individual exposure variation, especially the floor of the dwelling, is an important limitation of the present study, and may be prone to Berkson error, as subjects may be differentially exposed due to housing characteristics, ventilation practices, and/or time spent at home. The Berkson error model refers to random misclassification that results in little to no bias in the measurement, whereas classical non-differential measurement error tends to bias the risk estimates toward the null (33–35). Yet, for the same reasons as above, we did not recontact patients and their relatives to collect information on the height of the story, nor the patients’ lifetime residential history to assess past exposures.

While age distribution, histology, and stage at diagnosis were similar to national data, survival rates of patients in our study overall (i.e., survival rates of 77 and 38%, at 1 and 5 years, respectively), were higher than the national average (i.e., 47 and 20% at 1 and 5 years, respectively), at the same time (36). The comprehensive Cancer Center Léon Berard being a regional oncology reference center, it recruits many patients in relapse or in their later line of treatment, which may contribute to the increased survival observed. Moreover, cancer patients treated in institutions with high accrual volume of clinical trials present superior overall survival (37).

Socio-economic status, both at the individual level and area level, has been associated with survival in lung cancer patients (38, 39). In our study, area-level socio-economic status was not associated with mortality. Area-level socio-economic status has been suggested as a proxy for individual socio-economic status, in particular at smaller scales and for self-reported data (15, 40). Yet, estimates using area-level variables as surrogates of individual socio-economic status may be biased toward the null, partly explaining the observed lack of effect (41). However, as the analyses included other major prognostic factors, the observed lack of effect of socio-economic status on lung cancer survival is consistent with previous studies showing similar findings (42, 43).

Also, some prognostic factors were not available and therefore not included in the study, such as occupational exposure (44) or performance status, the latter playing a role in the choice of treatment and influencing the prognosis of patients (45).

Finally, a limitation of the model used is its inability to assess a time-dependent effect on categorical variables when all patients in one group experienced the event or a censoring before the end of the observation period. The choice of a 5-year maximum follow-up made it possible to overcome this limitation as not all patients in a group had yet experienced the event at this point.

In conclusion, lung cancer patient’s survival was not significantly associated with residential radon exposure in our study. Given the proportion of patients residing in areas with medium-to-high geogenic radon potential in the present study, close to the situation of the general population in the study area, further research should perform prospective indoor radon measurements in patients diagnosed with lung cancer to better understand the impact of indoor radon exposure on patient survival and second primary lung cancer as well as to implement mitigation and prevention strategies in dwellings to reduce indoor radon levels and exposure of patients and their relatives. Moreover, future studies should consider the patients’ complete residential history and collect relevant housing characteristics.

## Data availability statement

The original contributions presented in the study are included in the article/Supplementary material, further inquiries can be directed to the corresponding author.

## Ethics statement

The studies involving humans were approved by Center Léon Berard Committee of Research Ethics (reference 2022-003). The studies were conducted in accordance with the local legislation and institutional requirements. Written informed consent for participation was not required from the participants or the participants’ legal guardians/next of kin in accordance with the national legislation and institutional requirements.

## Author contributions

JD: Formal analysis, Investigation, Methodology, Validation, Visualization, Writing – original draft, Writing – review & editing. OP: Data curation, Writing – review & editing. CC: Formal analysis, Methodology, Visualization, Writing – original draft, Writing – review & editing. HN: Writing – review & editing. TC: Writing – review & editing. LG: Writing – review & editing. NF: Writing – review & editing. EB: Data curation, Writing – review & editing. JF: Writing – review & editing. FF: Writing – review & editing. AS: Writing – review & editing. E-MN: Writing – review & editing. PS: Writing – review & editing. MT: Writing – review & editing. MB: Writing – review & editing. FG: Writing – review & editing. VA: Writing – review & editing. MP: Writing – review & editing. BC: Writing – review & editing. BF: Conceptualization, Validation, Writing – original draft, Writing – review & editing.
