# Supplementary material for: Survival of bronchopulmonary cancers according to radon exposure
Source: Front Public Health. 2024 Jan 24;11:1306455. doi: 10.3389/fpubh.2023.1306455 (PMC10847230; doi:10.3389/fpubh.2023.1306455)
Supplement: Supplementary file 1 [file Table_1.DOCX]

Supplementary Material

**Supplementary Table 1. TEST OF COX’S PROPORTIONAL HAZARDS ASSUMPTION FOR GLOBAL SURVIVAL OF LUNG CANCER PATIENTS**

|  | chisq | df | p-value |
| --- | --- | --- | --- |
| **Potential radon categories** | 1.637 | 2 | 0.4411 |
| **Age at diagnosis** | 1.088 | 1 | 0.2968 |
| **Sex** | 1.599 | 1 | 0.2060 |
| **Area-level living standard** | 1.852 | 1 | 0.1736 |
| **Smoking status** | 4.379 | 2 | 0.1120 |
| **Histologic type** | 3.344 | 2 | 0.1878 |
| **Tumoral stages at diagnosis** | 12.929 | 3 | 0.0048 |
| **Mutations** | 0.789 | 2 | 0.6741 |
| **GLOBAL** | 32.994 | 14 | 0.0029 |

*Chisq: Chi-square test; df : degrees of freedom*

**
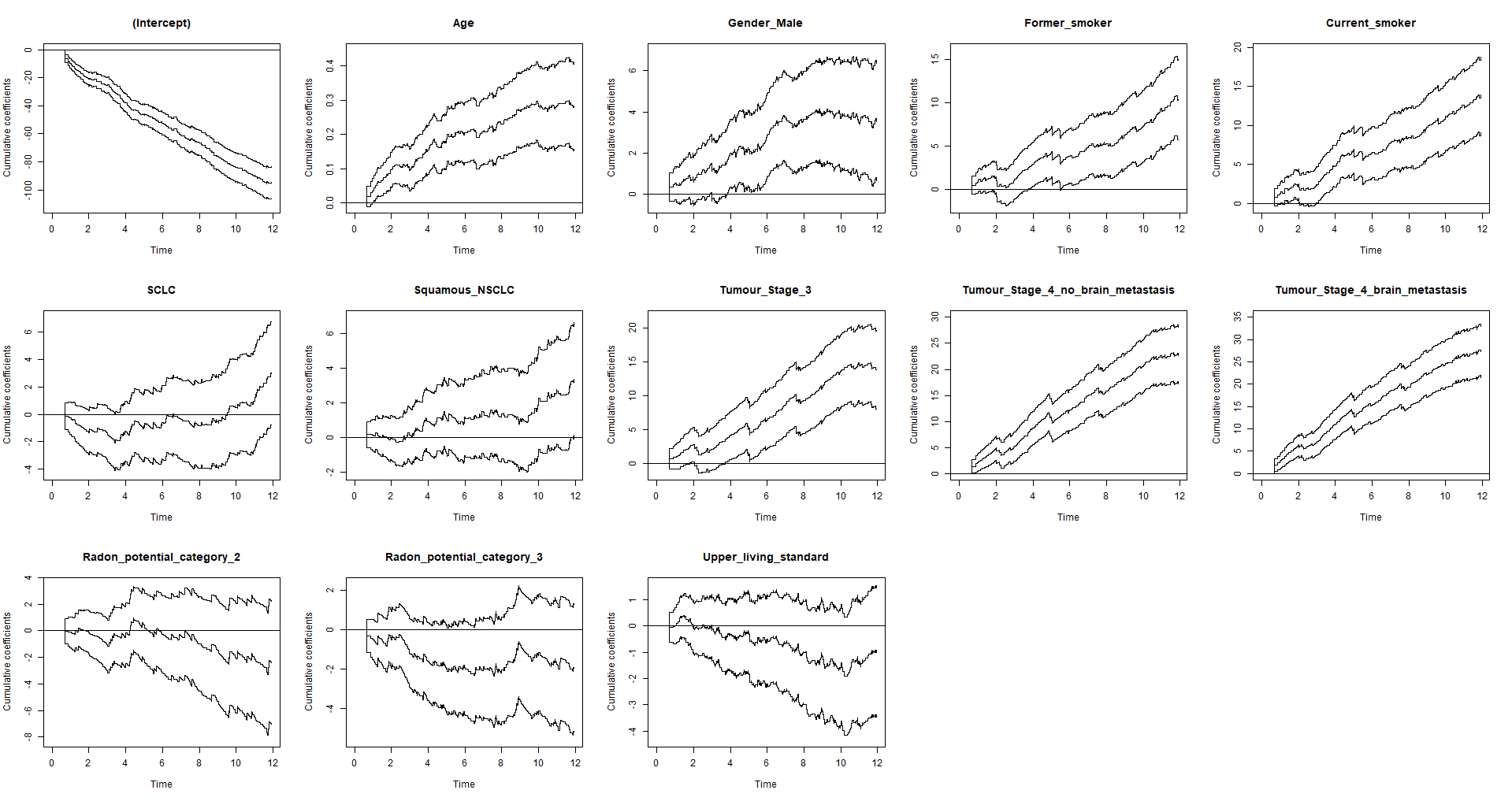
Supplementary Figure 2-A. ESTIMATION OF TIME-VARYING EFFECTS IN THE MULTIVARIABLE MODEL WITH ALL COVARIATES UP TO ONE YEAR.**

*At one year of follow-up, the effects of the following covariates were considered as time-varying: tumoral stage 3 and small cell histologic type. Other covariates risks were considered as constant over time.*

**Supplementary Figure** **2-B**. **ESTIMATION OF TIME-VARYING EFFECTS IN THE MULTIVARIABLE MODEL WITH ALL COVARIATES UP TO THREE YEARS.**


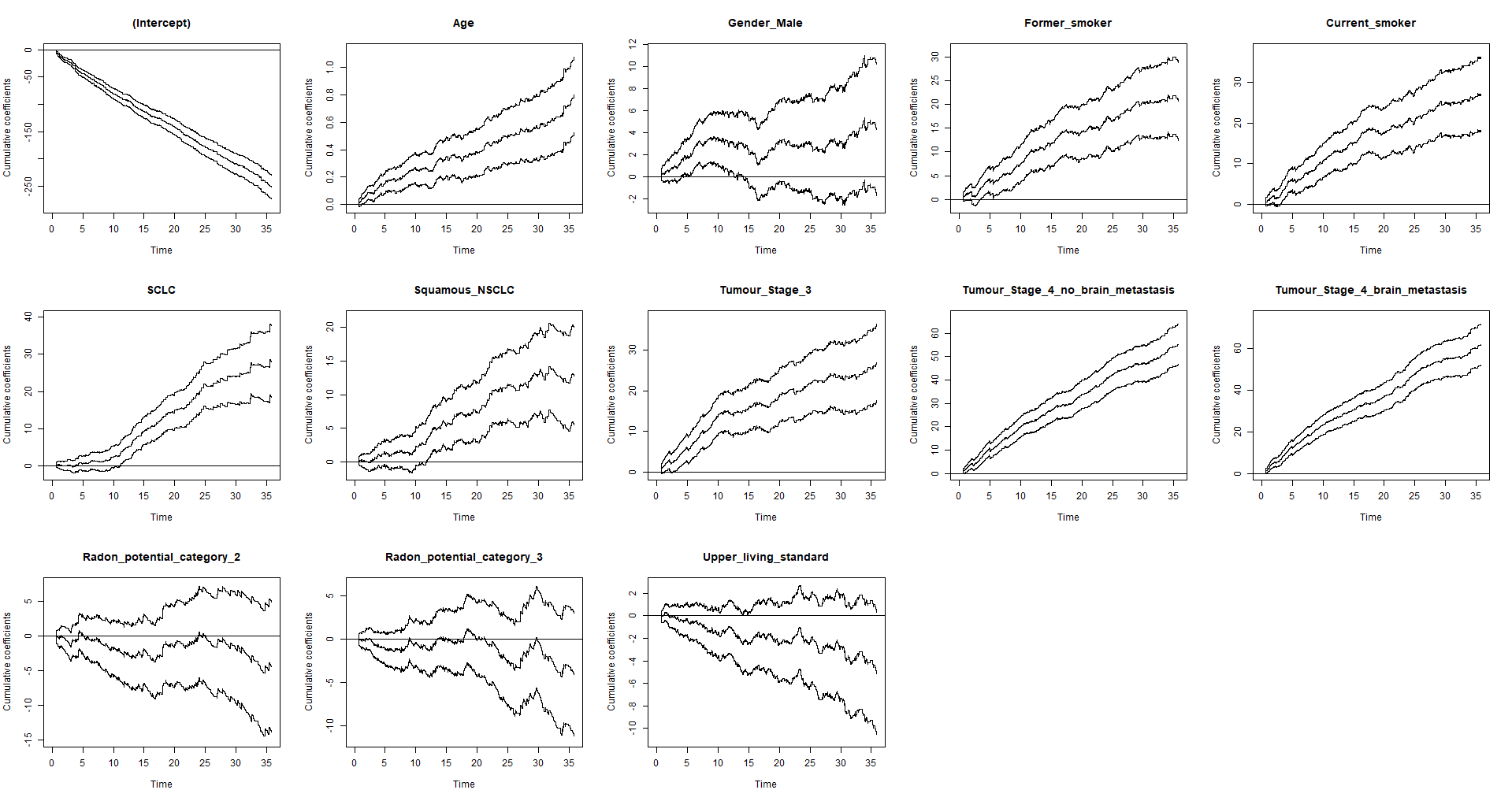
*At three years of follow-up, all effects were considered as constant.*

**Supplementary Figure** **2-C.** **ESTIMATION OF TIME-VARYING EFFECTS IN THE MULTIVARIABLE MODEL WITH ALL COVARIATES UP TO FIVE YEARS.**


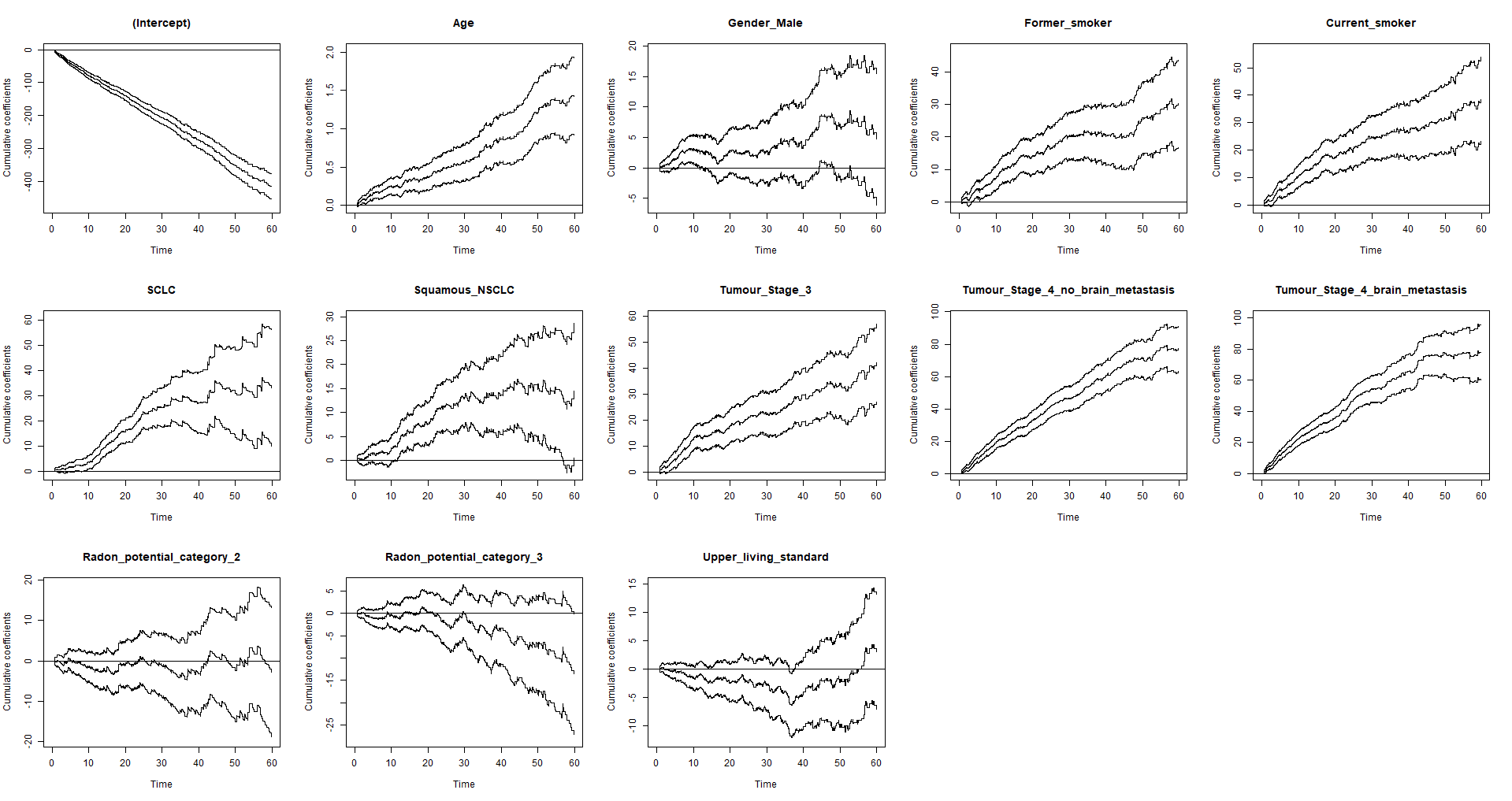
*At five years of follow-up, the effects of the following covariates were considered as time-varying: tumoral stage IV, with and without brain metastasis, and living standard upper the median. Other covariates risks were considered as constant over time.*

**Supplementary** **Table 3. P-VALUES OF THE CRAMER VON-MISES TEST FOR THE ASSOCIATION OF A FIXED EFFECT ON COVARIATES IN THE BACKWARD PROCEDURE**

|  | **1 year of follow-up** | **3 years of follow-up** | **5 years of follow-up** |
| --- | --- | --- | --- |
|  | **p-value*** | **p-value*** | **p-value*** |
| **Age at diagnosis (per year)** | 0.065 | 0.304 | 0.491 |
| **Gender** |  |  |  |
| Female | - | - | - |
| Male | 0.062 | 0.406 | 0.500 |
| **Area-level living standard** |  |  |  |
| Below the regional median | - | - | - |
| Above the regional median | 0.566 | 0.607 | **0.022** |
| **Smoking status at diagnosis** |  |  |  |
| Never smoker | - | - | - |
| Former smoker | 0.087 | 0.142 | 0.267 |
| Current smoker | 0.656 | 0.607 | 0.511 |
| **Histologic type** |  |  |  |
| Non-squamous NSCLC | - | - | - |
| SCLC | **0.002** | 0.162 | 0.213 |
| Squamous NSCLC | 0.148 | 0.389 | 0.155 |
| **Stage at diagnosis** |  |  |  |
| I/II | - | - | - |
| III | **0.029** | 0.490 | 0.452 |
| IV without brain metastasis | 0.326 | 0.127 | **0.028** |
| IV with brain metastasis | 0.122 | 0.254 | **0.001** |
| **Potential radon categories** |  |  |  |
| Category 1 | - | - | - |
| Category 2 | 0.638 | 0.532 | 0.724 |
| Category 3 | 0.150 | 0.431 | 0.238 |

**For each covariate, the last p-value for inclusion as time-varying effect in the backward procedure is displayed (no available p-value afterwards).*
